# Author Correction: Deglacial water-table decline in Southern California recorded by noble gas isotopes

**DOI:** 10.1038/s41467-021-24923-x

**Published:** 2021-08-03

**Authors:** Alan M. Seltzer, Jessica Ng, Wesley R. Danskin, Justin T. Kulongoski, Riley S. Gannon, Martin Stute, Jeffrey P. Severinghaus

**Affiliations:** 1 Scripps Institution of Oceanography, University of California, San Diego, 9500 Gilman Drive, La Jolla, CA 92093 USA; 2 California Water Science Center, United States Geological Survey, 4165 Spruance Road, San Diego, CA 92101 USA; 3 Lamont-Doherty Earth Observatory of Columbia University, 61 Route 9W, Palisades, NY 10964 USA; 4 Barnard College, 3009 Broadway, New York, NY 10027 USA

**Keywords:** Palaeoclimate, Hydrology

Correction to: *Nature Communications* 10.1038/s41467-019-13693-2, published online 16 December 2019.

The original version of the Supplementary Information associated with this Article included an incorrect Supplementary Data file, in which, the second sheet of the Excel workbook had misaligned sample IDs with corresponding data. The HTML has been updated to include a corrected version of Supplementary Data; the original incorrect version of Supplementary Data can be found as Supplementary Information associated with this Correction.

## Supplementary information

Supplementary Information

