# Supplementary material for: Deglacial water-table decline in Southern California recorded by noble gas isotopes
Source: Nat Commun. 2019 Dec 16;10:5739. doi: 10.1038/s41467-019-13693-2 (PMC6915717; doi:10.1038/s41467-019-13693-2)
Supplement: Supplementary file 1 — Supplementary Information [file 41467_2019_13693_MOESM1_ESM.pdf]

Supplementary Information for:

Deglacial water-table decline in Southern California recorded by noble gas isotopes

by Seltzer, A.M. et al.

Supplementary Note 1. Details on analytical corrections, normalization, and precision

In this study, we report all measured isotope ratios as delta values with respect to atmospheric air, such that:

$$\delta = \frac{\left(\frac{h}{l}\right)_{smp}}{\left(\frac{h}{l}\right)_{atm}} - 1 \qquad (1)$$

where δ is given in ‰ or per meg and *h* and *l* are heavy and light isotopes in a sample (smp) or atmospheric air (atm). All samples and atmospheric standards were analyzed against a common reference gas using a Thermo-Finnigan MAT 253 dual-inlet isotope ratio mass spectrometer. We define raw sample or atmospheric air measurements relative to the common reference gas (ref) as δ', where:

$$\delta'_{smp} = \frac{\left(\frac{h}{l}\right)_{smp}}{\left(\frac{h}{l}\right)_{ref}} - 1 \qquad (2)$$

and

$$\delta'_{atm} = \frac{\left(\frac{h}{l}\right)_{atm}}{\left(\frac{h}{l}\right)_{ref}} - 1 \qquad (3)$$

Normalized sample delta values were then calculated as follows:

$$\delta = \frac{\delta'_{smp}+1}{\delta'_{atm}+1} - 1 \qquad (4)$$

We report analytical precision based on the pooled standard deviations of replicate samples (σ_{pld}) for each individual measured isotope ratio. We estimate the absolute error of atmosphere-normalized measurements by accounting for the standard error of repeated δ'_{atm} measurements as well as and uncertainty in the various corrections made to δ'_{smp}. Over the course of this study, two campaigns (A and B) were carried out, separated by replacement of the ion-source filament in June 2018. Supplementary Table S1 lists the heavy and light isotopes measured for each of these ratios as well as the campaign A and B pooled standard deviations (σ_{pld}), the standard error of atmospheric air measurements against the working standard gas (SE_{atm}), the standard error of standard aliquot tests (SE_{ext}), which were carried out to correct for any fractionation induced by extraction and/or purification as described in detail in Seltzer et al. (2019), and the absolute error associated with chemical slope corrections (SE_{CS}), which we assume is equal to 10% of the average correction magnitude. The chemical slope correction is a routine correction [1], which we make to Kr and Xe isotope ratios due to sample and standard differences in Ar/Kr and Ar/Xe ratios and was determined empirically through tests carried out nine times between August 2017 and September 2018, in which pure Ar was added to aliquots of standard gas in different proportions to test for apparent isotopic sensitivity to Ar/Kr and Ar/Xe ratios.

Supplementary Table 1.

Isotope ratios measured in this study and associated uncertainty metrics. Heavy and light isotope masses (m_h and m_l) are given in amu; uncertainty metrics are given in per meg.

Ratio	m_h	m_l	$\sigma_{pld,A}$	$\sigma_{pld,B}$	SE_{atm}	SE_{ext}	SE_{CS}
$\delta^{40}/_{36}Ar$	40	36	19	19	3	5	n/a
$\delta^{38}/_{36}Ar$	38	36	21	12	5	8	n/a
$\delta^{86}/_{82}Kr$	86	82	17	24	10	4	1
$\delta^{86}/_{83}Kr$	86	83	15	27	7	5	1
$\delta^{86}/_{84}Kr$	86	84	12	23	4	11	5
$\delta^{136}/_{129}Xe$	136	129	31	46	17	9	2
$\delta^{134}/_{129}Xe$	134	129	28	52	18	9	3
$\delta^{132}/_{129}Xe$	132	129	19	28	10	6	1

Formally, the standard error of a given delta value measured in *N* replicate samples is given by the quadrature sum of measurement precision, divided by $\sqrt{N}$, and the standard errors of atmospheric air measurements and analytical corrections:

$$SE_{tot} = \sqrt{\left(\left(\frac{\sigma_{pld}}{\sqrt{N}\cdot(\delta'_{smp}+1)}\right)^2 + \left(\frac{SE_{atm}}{(\delta'_{atm}+1)}\right)^2\right)\cdot|\delta+1| + {SE_{CS}}^2 + {SE_{ext}}^2} \qquad (5a)$$

For a single sample (*N*=1), SE_{tot} represents the absolute uncertainty of δ relative to the atmosphere, whereas σ_{pld} represents measurement reproducibility. In this study (e.g. Figs. 3 and 4), individual samples are all treated independently in inverse modeling for water-table depth, with Monte Carlo simulations carried out separately based on each term in Supplementary Equation 5. In practice, we make use of the approximate additivity of delta values to calculate SE_{tot}, valid to ~0.1 per meg amu^{-1}, for each isotope ratio measured over an entire analytical campaign, which is needed in Supplementary Equations 6 and 7 below. This approximation is given as follows:

$$SE_{tot} \approx \sqrt{\left(\frac{\sigma_{pld}}{\sqrt{N}}\right)^2 + {SE_{atm}}^2 + {SE_{CS}}^2 + {SE_{ext}}^2} \qquad (5b)$$

Bulk Kr and Xe concentrations were determined by first normalizing measured δKr/Ar and δXe/Ar ratios to atmospheric ratios [2] to determine absolute Xe/Ar and Kr/Ar in samples and then multiplying Xe/Ar and Kr/Ar by the measured dissolved Ar concentration. Kr/Ar and Xe/Ar were assumed equal to the measured ratios of $^{84}Kr/^{40}Ar$ and $^{132}Xe/^{40}Ar$, respectively. In campaigns A and B, dissolved Ar, Kr, and Xe concentration pooled standard deviations were ±~0.7% and ~0.4%, respectively.

We make use of the linear mass dependence of soil air fractionation processes [3] and solubility fractionation [4] for Kr and Xe isotopes to define the mass difference-normalized, error-weighted mean parameters δ*Kr and δ*Xe in per meg amu^{-1}:

$$\delta^*\text{Kr} = \frac{\frac{\delta^{86}_{82}Kr}{4\text{ amu}}\left(\frac{4\text{ amu}}{SE_{tot,\frac{86}{82}}}\right)^2 + \frac{\delta^{86}_{83}Kr}{3\text{ amu}}\left(\frac{3\text{ amu}}{SE_{tot,\frac{86}{83}}}\right)^2 + \frac{\delta^{86}_{84}Kr}{2\text{ amu}}\left(\frac{2\text{ amu}}{SE_{tot,\frac{86}{84}}}\right)^2}{\left(\frac{4\text{ amu}}{SE_{tot,\frac{86}{82}}}\right)^2 + \left(\frac{3\text{ amu}}{SE_{tot,\frac{86}{83}}}\right)^2 + \left(\frac{2\text{ amu}}{SE_{tot,\frac{86}{84}}}\right)^2} \tag{6}$$

and

$$\delta^*\text{Xe} = \frac{\frac{\delta^{136}_{129}Xe}{7\text{ amu}}\left(\frac{7\text{ amu}}{SE_{tot,\frac{136}{129}}}\right)^2 + \frac{\delta^{134}_{129}Xe}{5\text{ amu}}\left(\frac{5\text{ amu}}{SE_{tot,\frac{134}{129}}}\right)^2 + \frac{\delta^{132}_{129}Xe}{3\text{ amu}}\left(\frac{3\text{ amu}}{SE_{tot,\frac{132}{129}}}\right)^2}{\left(\frac{7\text{ amu}}{SE_{tot,\frac{136}{129}}}\right)^2 + \left(\frac{5\text{ amu}}{SE_{tot,\frac{134}{129}}}\right)^2 + \left(\frac{3\text{ amu}}{SE_{tot,\frac{132}{129}}}\right)^2} \tag{7}$$

In terms of these mass difference-normalized variables, the analytical precision of campaigns A and B (i.e. $\sigma_{pld,A}$ and $\sigma_{pld,B}$, respectively) was 3.6 and 5.2 per meg amu^{-1} for δ*Xe and 3.5 and 5.0 per meg amu^{-1} for δ*Kr, respectively.

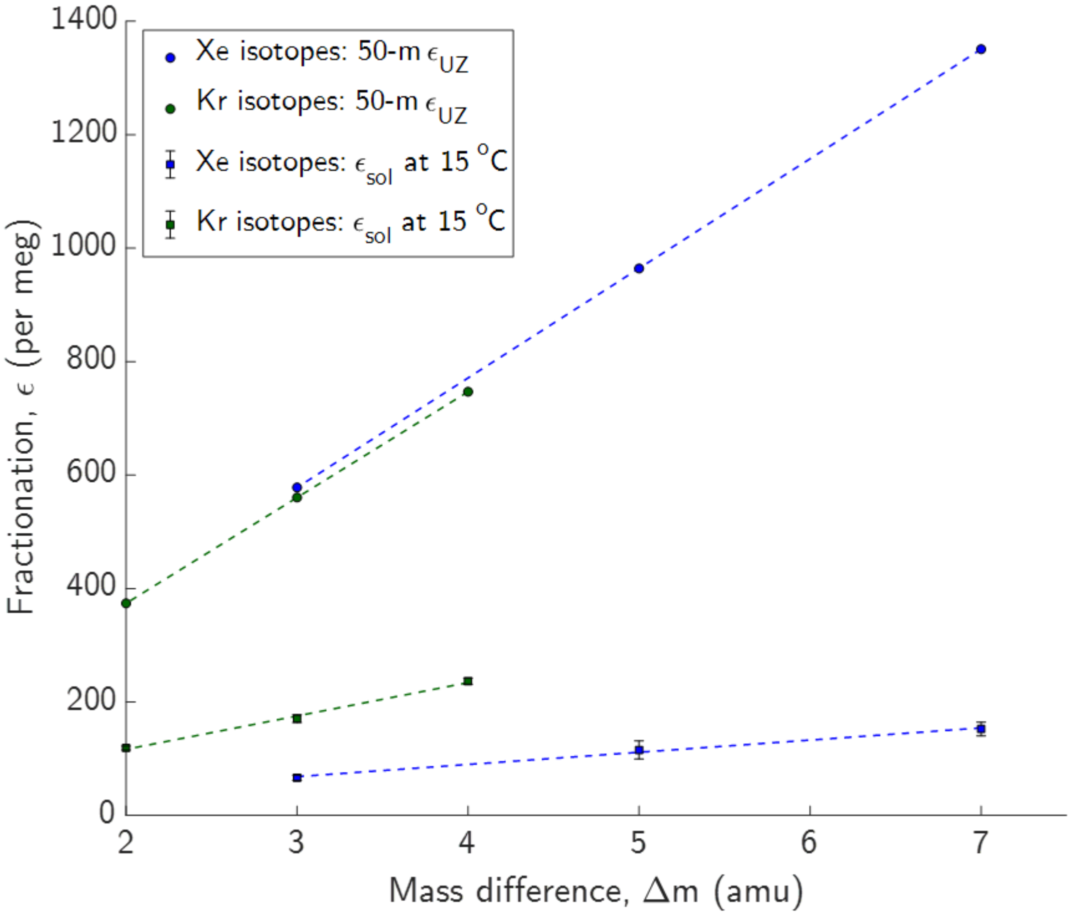

Supplementary Figure 1: Linear mass dependence of Xe and Kr isotope ratios for modeled steady-state soil air fractionation (at 50 m below the land surface) and for empirically-determined solubility fractionation in fresh water (at 15 ^{o}C).

The assumption of linear mass dependence can be tested by comparing modeled and measured ratios to isotopic mass difference (Supplementary Figure 1), for integrated soil air fractionation (ε_{UZ}, which consists of gravitational settling, thermal diffusion, and water-vapor flux fractionation) and solubility fractionation (ε_{sol}) . In Supplementary Figure 1, we plot ε_{UZ} and ε_{sol} vs isotopic mass difference (Δm) for the steady-state soil air fractionation model (e.g. as shown for δ*Xe in Fig. 1) for individual Kr and Xe isotope ratios. Modeled UZ air fractionation (at 50 m) is shown in Supplementary Figure 1. The model assumes soil air fractionation occurs due to gravitational settling, thermal diffusion (due to a 30 ^{o}C km^{-1} geothermal gradient) and water-vapor flux fractionation driven by mean annual surface air relative humidity and temperature of 70% and 20 ^{o}C, respectively. The full equations governing these three fractionation processes are presented in Supplementary Note 2 (Inverse Model Overview). In Supplementary Figure 1, each marker indicates the fractionation of an individual isotope ratio with mass difference Δm (e.g. 7 amu for $\delta^{136}/_{129}$Xe). We suggest that this linearity justifies the use of simple δ*Xe and δ*Kr notation for presentation purposes. For the full inverse model (described in Supplementary Note 2), however, each isotope ratio is treated independently, and no assumption of linear mass dependence is needed.

Supplementary Note 2. Inverse Model Overview

In this study, two separate inverse models were coupled in an iterative loop and constrained by measurements made in each sample. One model solves for mean annual surface temperature (MAST) and is constrained by noble gas concentrations and the other solves for water-table depth (WTD) and is constrained by noble gas isotope ratios.

The first inverse model estimates dissolution parameters (recharge temperature and excess air) from dissolved concentrations, employing an adapted version of the widely used Closed-system Equilibration (CE) model [5,6]. This model is identical to the CE model except that instead of assuming that unsaturated zone (UZ) air at the water table is unfractionated from atmospheric noble gas composition, it assumes Ar/air, Kr/air, and Xe/air ratios are fractionated by gravitational settling, thermal diffusion, and water-vapor flux fractionation. The magnitude of total UZ air fractionation for a given elemental or isotopic ratio is $\varepsilon_{UZ.}$ This model requires knowledge of surface pressure, which we prescribe based on elevation. It yields estimates of recharge temperature (T, ^{o}C), initially entrapped air volumetric ratio (A, volume air/volume water), and dissolution amount (F, final air volume / initial air volume).

The second inverse model concerns UZ fractionation (ε_{UZ}), yielding estimates of the two free parameters governing ε_{UZ}: WTD (which sets the gravitational settling signal) and surface water-vapor mole fraction (X_{H2O}, which sets the water-vapor flux fractionation). This model requires the known geothermal gradient to determine thermal diffusion and water-vapor flux fractionations between unfractionated surface air and UZ air above the water table. The geothermal gradient at each site was either measured (for San Diego samples by the USGS during well installation) or taken from nearby observations and was assumed to be constant in time.

Formally, the first model solves for a dissolved concentration of a gas X (Ar, Kr, or Xe), *[X]*, in terms of T, A, F following the formulation of Aeschbach-Hertig et al. (2008):

$$[X] = [X]_{eq}(T, P, \varepsilon_{UZ}) * \left[1 + \frac{(1-F)A*H(T)}{1+FA*H(T)}\right] \quad (8)$$

where the subscript "eq" indicates solubility equilibrium and H is the Henry coefficient ($H \equiv [X]_{air}/[X]_{eq}$). Here, solubility equilibrium refers to specifically to an equilibrium between groundwater dissolved gases and overlying UZ air (importantly not atmospheric air) at the water table. The solubility concentrations (and Henry coefficients) are prescribed using the recently redetermined noble gas solubility functions of Jenkins et al. (2019). With knowledge of T, A, and F, ε_{sol} and ε_{EA} can be determined from the known isotopic solubility functions [4] and extension of the CE model to isotope ratios, respectively. We note that Seltzer et al. (2019) used newly measured Kr and Xe isotopic solubility and diffusivity fractionation factors to demonstrate that ε_{EA} is insensitive to the choice of excess air model at the single per meg amu^{-1} level. Extending S6 to determine ε_{EA} for the CE model results in:

$$\varepsilon_{EA} = \frac{(1+AH_g)(1+AF_{CE}H_g\alpha_{sol})}{(1+AH_g\alpha_{sol})(1+AF_{CE}H_g)} - 1 \quad (9)$$

where H_g is the Henry coefficient for either bulk Xe or Kr and $\alpha_{sol} = 10^{-6}\varepsilon_{sol} + 1$ (if ε_{sol} is given in per meg). Note that multiplying Supplementary Equation 9 (or any subsequent equation concerning ε values) by 10^6 will give values in per meg.

The second model computes ε_{UZ} for individual isotopic or elemental ratios as a function of WTD, X_{H2O}, and geothermal gradient (Γ). It assumes that steady-state fractionation of UZ air above the water table is due to gravitational settling, thermal diffusion, and water-vapor flux fractionation, such that:

$$\varepsilon_{UZ} = \varepsilon_{grav} + \varepsilon_{H2O} + \varepsilon_{therm} \quad (10)$$

Gravitational settling fractionation [8], ε_{grav}, is parameterized by:

$$\varepsilon_{grav} = e^{(WTD*\Delta m*g)/(RT)} - 1 \quad (11)$$

where R is the ideal gas constant (8.314 m^3 Pa K^{-1} mol^{-1}), T is UZ air temperature (K), g is gravitational acceleration (9.8 m s^{-2}), and WTD and Δm are given in m and in kg mol^{-1}, respectively. The parameterization for steady-state water-vapor flux fractionation [9], ε_{H2O}, is given by:

$$\varepsilon_{H2O} \approx \left(\frac{1-e_{sat}/P_{tot}}{1-\chi_{H2O}}\right)^{\frac{D_{h\text{-}H2O}}{D_{l\text{-}H2O}}-1} - 1 \quad (12)$$

where X_{H2O} is the mole fraction of water vapor in mean surface air, e_{sat} is saturation vapor pressure in UZ air above the water table, P_{tot} is total UZ air pressure above the water table, and $\frac{D_{h\text{-}H2O}}{D_{l\text{-}H2O}}$ is the binary diffusivity ratio of heavy and light gases or isotopes against water vapor [10]. Thermal diffusion fractionation is given by:

$$\varepsilon_{therm} = -\Omega*\Gamma*WTD \quad (13)$$

where Γ is the geothermal gradient (in °C m^{-1}) and Ω is thermal diffusion sensitivity for a given gas or isotope ratio [11,12].

As illustrated in Supplementary Figure 2, the two inverse models are coupled in an iterative algorithm to estimate WTD and T from measurements of Kr and Xe isotope ratios and bulk noble gas concentrations from a single sample, with geothermal gradient and elevation prescribed. MAST can then be determined by subtracting Γ·WTD from T, thus removing the contribution of geothermal heat from the estimated recharge temperature (at WTD). The algorithm begins by first assuming no fractionation of elemental noble gas ratios in UZ air (i.e. $\varepsilon_{UZ} = 0$) and inverts the CE model via non-linear least squares (Levenberg-Marquardt algorithm) to find initial estimates of T, A, and F constrained by noble gas concentration measurements. Then, ε_{EA} for each Kr and Xe isotope ratio is computed via Supplementary Equation 9 and ε_{UZ} for each isotope ratio is estimated by subtracting ($\varepsilon_{EA} + \varepsilon_{sol}$) from δ. These six Kr and Xe isotope ratio estimates of ε_{UZ} are used to invert the UZ air fractionation model (i.e. Supplementary Equation 10), again via non-linear least squares, to estimate WTD and X_{H2O}. Note that because Kr and Xe isotopes are very weakly sensitive to water-vapor flux fractionation, estimates of X_{H2O} are poorly constrained and the least-squares solver is bounded to a surface-air relative humidity range between 25 and 100%. Next, using these parameters, ε_{UZ} is calculated for Ar/air, Kr/air, and Xe/air and the CE model is again inverted, this time assuming a fractionated UZ air composition above the water table to get a new set of T, A, and F estimates. These new estimates feed back into the UZ air model, and this back-and-forth process is repeated until WTD and T values converge within 50 cm and 0.2 °C, respectively.

Ultimately, for each groundwater sample this algorithm is carried out in 1000 separate Monte Carlo simulations, in which normally distributed random numbers with standard deviations equal to SE_{tot} are added to each measured isotope ratio and gas concentration. The results are thus 1000-element distributions of estimated WTD and T_{surf}, which provide uncertainties on these parameters. For tritiated (young) water samples, where ^{40}K-derived radiogenic ^{40}Ar can be neglected, Ar isotope ratios were also used to constrain the inverse model.

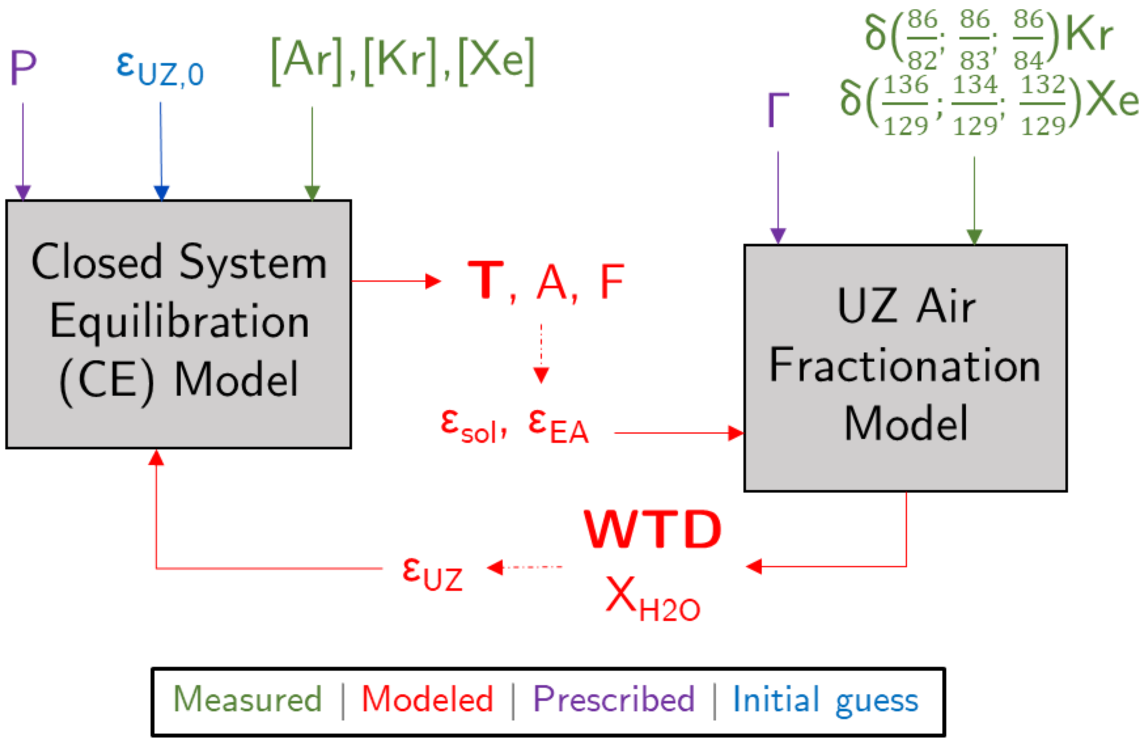

Supplementary Figure 2. Process diagram for iterative, coupled inverse modeling of surface temperature and WTD. The CE model is initially solved for T, A, and F based on the assumption of unfractionated WTD UZ air, constrained by measured concentrations and prescribed surface pressure. Using T, A, and F, ε_{sol} and ε_{EA} are determined for individual Kr and Xe isotope ratios. Then, measured Kr and Xe isotope ratios, prescribed Γ, and estimates of ε_{sol} and ε_{EA} are used to constrain the UZ air fractionation model to estimate WTD and X_{H2O}. In turn, ε_{UZ} for elemental ratios is determined (from WTD, Γ, and X_{H2O}), and the CE model is re-evaluated to produce updated estimates of T, A, and F. The soil air fractionation model is re-evaluated using updated ε_{sol} and ε_{EA} values. The iterative coupling of the two models proceeds until estimates of WTD and T converge to within 50 cm and 0.2 °C, respectively, for successive iterations. The entire process is repeated in 1000 Monte Carlo simulations, with random Gaussian error prescribed to each measured parameter in each simulation, to yield distributions of WTD and T from each groundwater sample. Finally, T is converted to MAST by subtracting Γ·WTD.

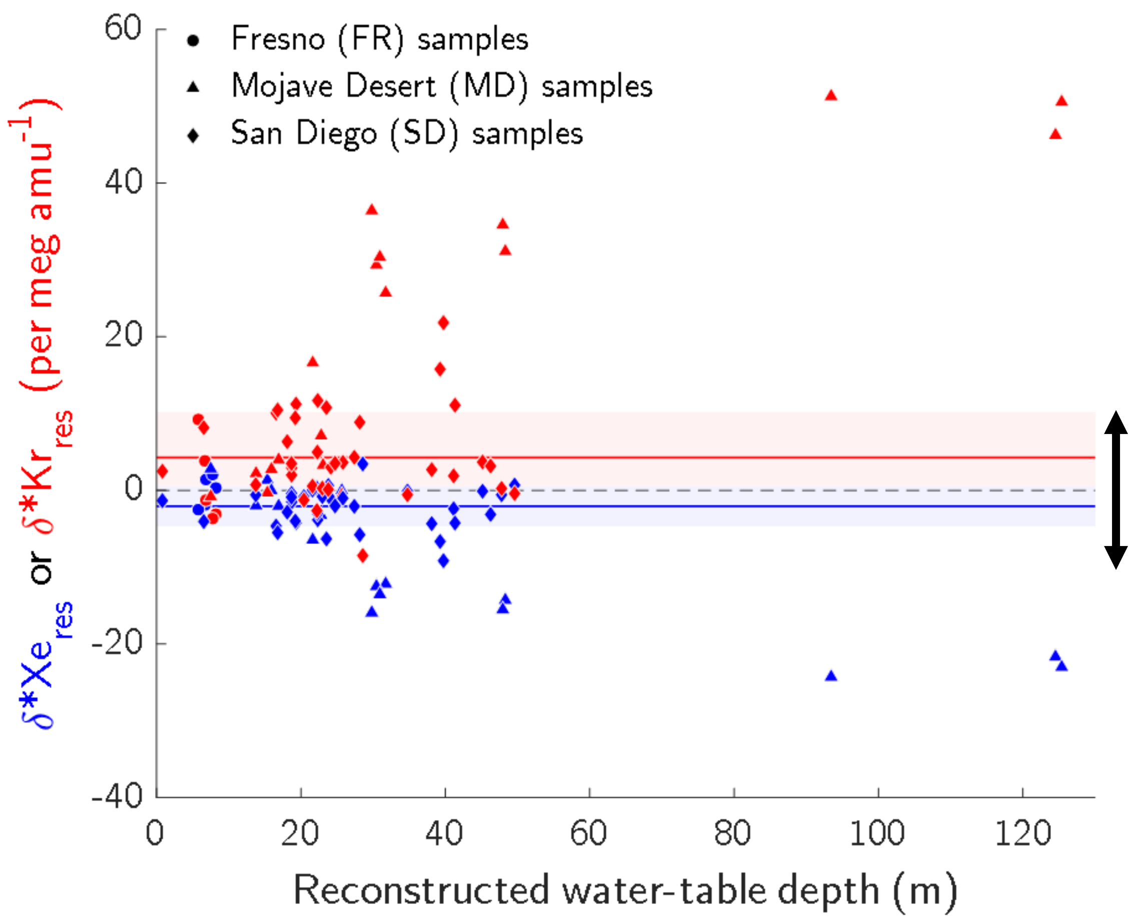

Supplementary Figure 3. Model-measurement Kr (red) and Xe (blue) isotopic residuals for each of the 58 samples analyzed in this study. Marker shapes indicate study area and solid lines indicate Fresno-and-San-Diego-only mean residuals. Shaded error regions indicate ±1-σ range of Fresno-and-San-Diego-only residuals. Arrows indicate ±2.5 meters of gravitational settling fractionation, for context.

The performance of the coupled-inverse model approach is investigated by looking at measurement-model residuals ($\delta_{res} = \delta_{measurement} - \delta_{model}$) for each individual sample analyzed in this study. In Supplementary Figure 3, Kr and Xe isotope residuals (δ^*Kr_{res} and δ^*Xe_{res}) are shown as a function of reconstructed WTD as well as study area. Across all Fresno and San Diego samples, there is no apparent trend of isotopic residuals with reconstructed WTD. Fresno

and San Diego mean δ^*Kr_{res} and δ^*Xe_{res} are 4 ± 6 and -2 ± 3 per meg amu^{-1} (1σ, N=40), respectively, each equivalent to one meter or less of gravitational settling fractionation. These small residuals add confidence to reconstructed WTDs in Fresno and San Diego samples at the several-meter scale. However, the fact that they are anti-correlated (positive Kr residuals and negative Xe residuals, on average) may indicate that a minor process is not captured by the model, perhaps causing maximum WTD biases for individual samples of several meters. Mojave Desert samples exhibit large, anti-correlated δ^*Kr_{res} and δ^*Xe_{res}, which appear to increase with reconstructed WTD up to maximum absolute values of 50 per meg amu^{-1}, equivalent to WTD biases of over 10 m for 90-125 m reconstructed WTDs. For these samples, δ^*Kr_{res} values are positive and δ^*Xe_{res} values are negative, likely indicating the failure of the model to capture a systematic process, perhaps the same process we suspect may play a minor role (at the single per meg amu^{-1} level on average) in the Fresno and San Diego samples. We therefore do not attempt to interpret reconstructed WTD in the Mojave Desert samples. In Supplementary Text Note 5, we propose and formally consider several physical processes that may explain the large isotopic residuals in the Mojave Desert samples.

Supplementary Note 3. Fresno Groundwater Study: Detailed Description

Five two-liter groundwater samples were collected from two wells located ~50 m apart at a site southeast of the city of Fresno, California. Two samples were collected from monitoring well 180-MW, which has a perforation interval from 65-68 meters below the land surface (mbls). Three samples were collected from the deeper supply well 180-1, which has a wider perforation interval from 125 to 186 mbls. The groundwater supplied to both wells comes from a regional unconfined aquifer system, which consists of fluvial deposits and generally flows from east to west [13].

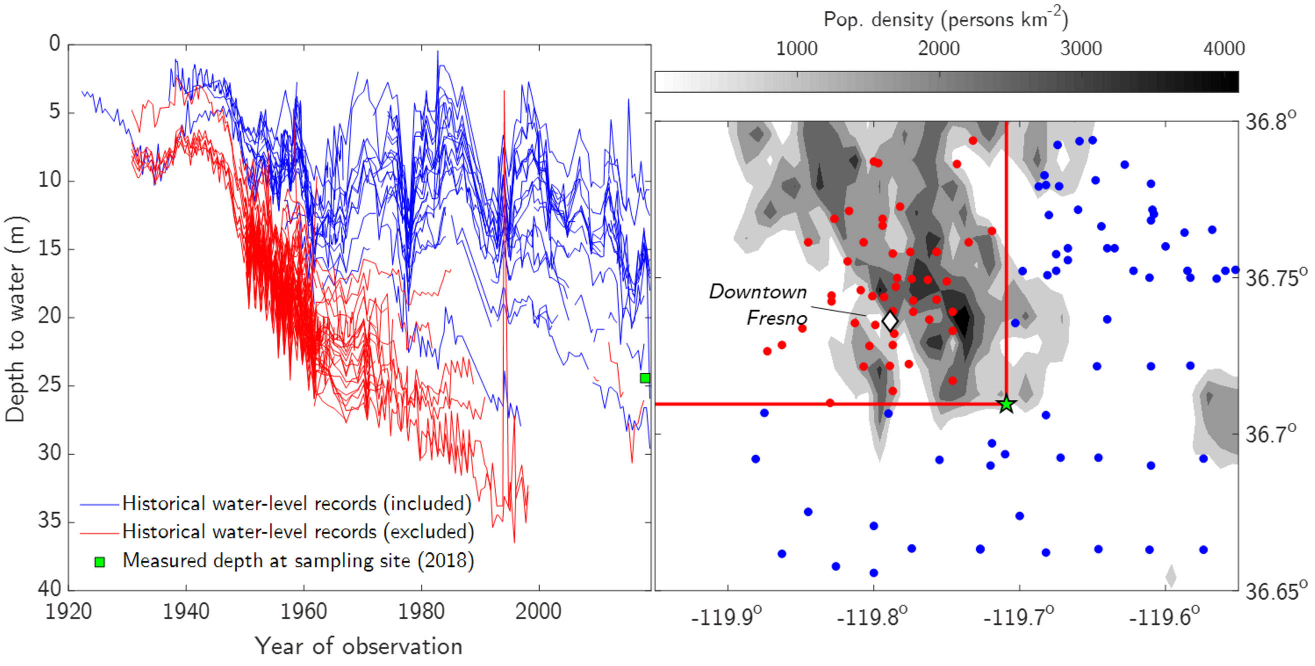

Supplementary Figure 4. Historical water-level records near Fresno, California, from the California Department of Water Resources Water Data Library. In the left panel, time series of observed water levels are shown for all sites within a 10 km radius of sampling location. Historical water-level observations to the northwest of sampling

location are shown in red and excluded from the noble gas-based water table depth comparisons (Figure 3). As shown in the right panel, these excluded observations are primarily from the population-dense Fresno city center, which presumably experienced more substantial mid-20th century groundwater abstraction than did the less populated portions of Fresno county. Locations of historical observations are superimposed on a map of year-2000 population density [14], and the sampling location is marked by a green star in the center of the map.

Although the city of Fresno was densely populated throughout the 20th century, the area directly surrounding these wells was historically agricultural until becoming more densely settled over the past ~30 years [13]. To estimate the local WTD at the time of recharge near these wells, we analyzed historical water-level records maintained by the California Department of Water resources (available online from https://data.cnra.ca.gov/dataset/periodic-groundwater-level-measurements) and considered all historical measurements from wells with a 10-km radius of the 180-1/180-MW sampling site. As shown in Supplementary Figure 4, the historical records appear to cluster into two distinct groups: one exhibiting substantial mid-20th century drawdown and another which appears to follow decadal-scale (natural) variability until the late 20th century. These groups exhibit a strong spatial dependence: all wells showing severe drawdown in the 1950s (specifically, with mean 1960s water levels below 15 m) lie within the densely populated Fresno city center to the northwest of the sampling site. We therefore separate these two groups along spatial criteria, such that all wells northwest of the 180-1/180-MW site belong to the first group (substantial mid-20th century groundwater abstraction), and all other wells (within the 10-km radius) belong to the second. These well groups are identified by the colors red and blue, respectively, in Supplementary Figure 4. Because the 180-1/180-MW site and its surrounding area was sparsely populated until the early 1990s, the most appropriate local WTD history is represented by the second group of wells, consisting of historical records from 76 individual wells.

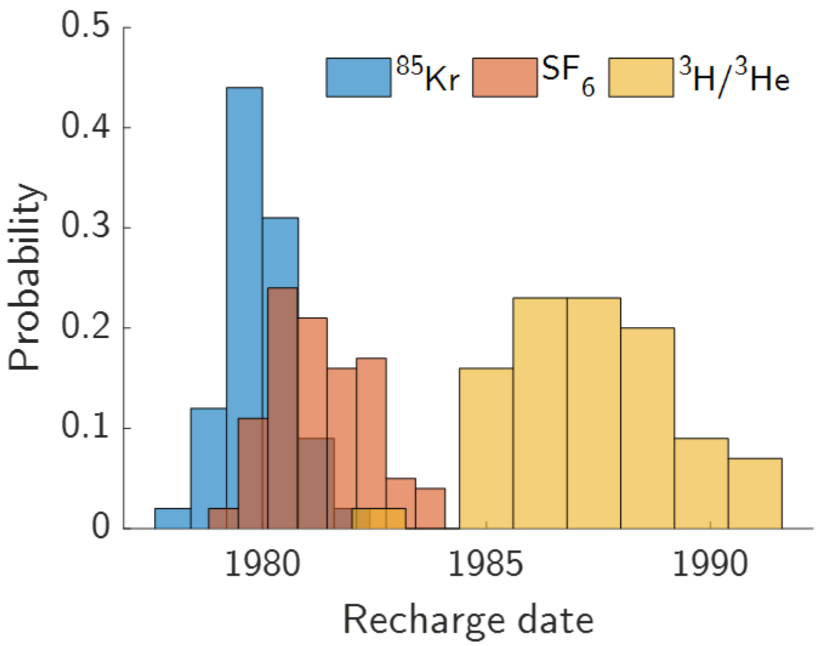

Supplementary Figure 5. Recharge date probability distributions for groundwater sampled from well 180-MW based on measured ^{85}Kr, SF_6, and $^3H/^3He$ ratios. In Figure 3, we assume a recharge date given by averaging the medians of each probability distribution. We assume this average recharge date, 1983, is accurate to within ± 5 years.

Groundwater samples from 180-MW were dated using three independent tracers: ^{85}Kr, $^{3}H/^{3}He$, and SF_6. In each case, piston flow age distributions were determined by carrying out 100 Monte Carlo simulations, as shown in Supplementary Figure 5, perturbing measurements and atmospheric histories with normally-distributed random numbers with standard deviations equal to analytical uncertainties. For ^{85}Kr, dissolved activity was measured at Argonne National Lab, and the European baseline atmospheric history [15] was used, interpolated to 0.1-year resolution and assigned a 5% 1σ uncertainty. For SF_6, dissolved measurements were made by gas chromatography at USGS Reston Groundwater Dating Lab. Historical atmospheric SF_6 measurements were taken from the National Oceanic and Atmospheric Administration's Niwot Ridge, Colorado, USA site (data available online from https://www.esrl.noaa.gov/gmd/dv/site/NWR.html) and UZ air SF_6 concentration at the time of recharge was determined based on the known SF_6 solubility [16], accounting for recharge temperature and excess air (via the CE model) based on bulk Ne, Ar, Kr, and Xe measurements made by the United States Geological Survey (USGS). USGS $^{3}He/^{4}He$ measurements and Lawrence Livermore National Lab ^{3}H measurements were used to determine $^{3}H/^{3}He$ piston flow ages assuming that the $^{3}He/^{4}He$ ratio in well 180-1 (which is ^{3}H dead) represents the background terrigenous helium isotope ratio. Piston flow age distributions from each age tracer yielded median recharge dates in the 1980s (Supplementary Figure 5): 1980.0 from ^{85}Kr, 1981.2 from SF_6, and 1987.6 from $^{3}H/^{3}He$. These piston flow ages imply an effective vertical velocity of ~1 m yr^{-1}, as the perforation interval of well 180-MW is ~40 m below the water table. Thus, the impact of dispersive mixing during sampling through the 3-m screened interval is unlikely to substantially bias recharge ages (Solomon et al., 1998). The close agreement of SF_6 and ^{85}Kr age distributions, which both appear older than the $^{3}H/^{3}He$ date, is in principle consistent with the well-known young bias of $^{3}H/^{3}He$ dating due to diffusive loss of radiogenic ^{3}He [18]. However, because the apparent vertical velocity is rather high, we conservatively determine the most probable recharge date to be the mean of the median ages from each tracer's distribution, 1983, and assign an uncertainty of ± 5 years. Because the WTD fluctuations exhibit decadal-scale variability (Fig. 3), the agreement of these three age tracers gives high confidence in comparing noble gas isotope-derived WTDs to early-to-mid 1980s historical water levels in the region.

Groundwater age in the deeper well (180-1), which has a large screen allowing for mixing of water of varying recharge age, was estimated based on SF_6, ^{85}Kr, $^{3}H/^{3}He$, ^{14}C, and ^{39}Ar. No ^{3}H or ^{85}Kr was detected (within error) and SF_6 concentrations were consistent with pre-1960s recharge, while ^{39}Ar and ^{14}C yielded piston flow ages of ~650 and ~6000 years respectively. Because ^{39}Ar has a ~20 times shorter half-life than ^{14}C, this is consistent with the groundwater from well 180-1 representing a mixture of water ranging in age from the mid Holocene to the last century. A more complete discussion of groundwater mixing and multi-tracer age estimation in these wells is the subject of another, ongoing study. Here we simply note that multiple age tracers confirm that 180-MW groundwater reflects 1980s recharge and that 180-1 groundwater reflects a mixture of mid-to-late Holocene recharge.

Supplementary Note 4. San Diego Groundwater Study: Detailed Description

Thirty-five two-liter groundwater samples were collected from a coastal aquifer system in San Diego, California. These samples were collected and analyzed from 23 monitoring wells pertaining to six separate multiple-depth, monitoring-well sites, which span a latitudinal range of ~13 km and longitudinal range of ~5 km (Figs. 4, S6). Each monitoring well has a narrow perforated interval (generally 6 m), which limits dispersive mixing during sampling. The six sites

are marked by the black polygons in Supplementary Figure 6. The coastal aquifer system is composed of a shallow, locally recharged, unconfined aquifer overlying a confined, regional aquifer composed of poorly consolidated marine sediment [19]. The regional aquifer is of primary interest to this study because the large spatial and temporal scales of UZ-groundwater exchange prior to confinement and intra-aquifer dispersive mixing integrate contemporaneous geochemical signals over a wide region. Indeed, the close agreement of the WTD reconstructions (Fig. 4) from regional-aquifer groundwater of similar recharge age (e.g. the clustering of 11 noble gas-derived WTD reconstructions near 20 meters from last glacial period samples across many well sites, Fig. 4) is consistent with the notion that deep, confined groundwater reflects integrated signals over a wide spatial range.

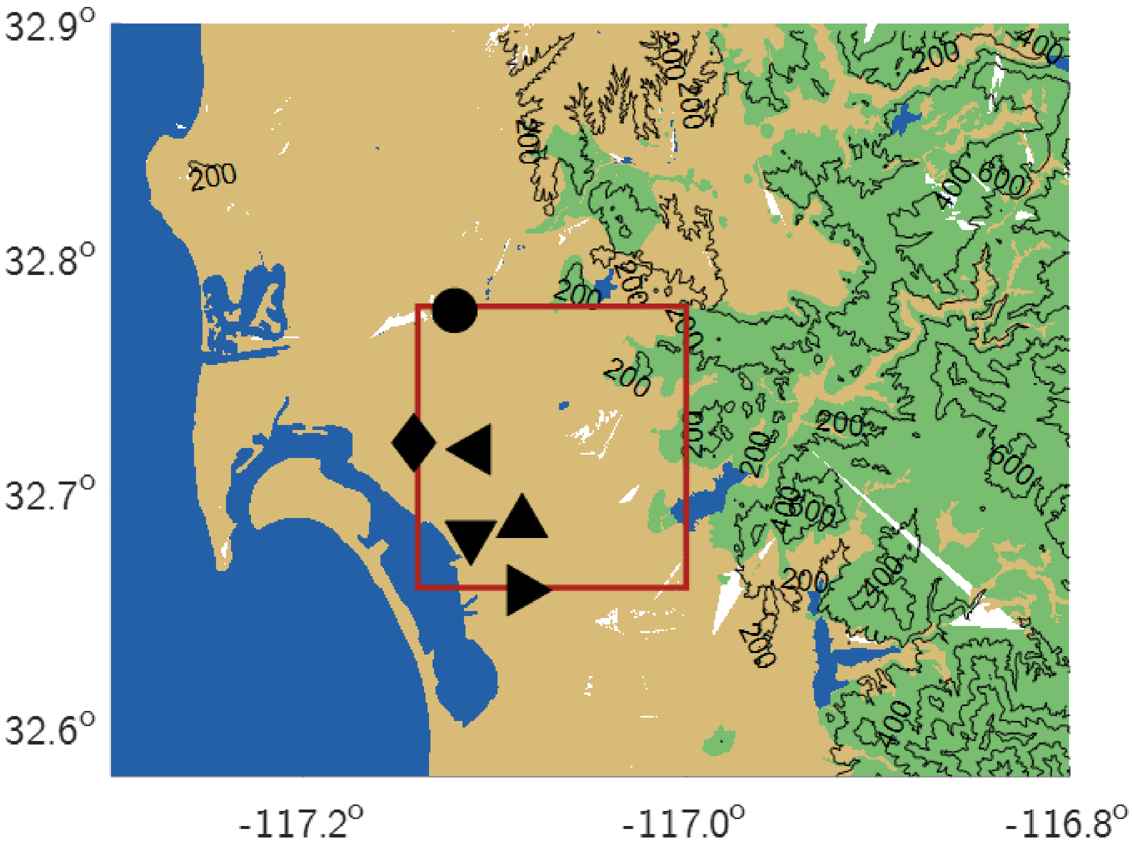

Supplementary Figure 6. San Diego study area. Six multiple-depth, monitoring-well sites are marked by black polygons. The assumed recharge area for deep, regional groundwater flow to these sites is indicated by the red box. Beige coloring indicates unconsolidated sediment, green indicates igneous or metamorphic rock, and blue indicates water. Elevation contours, spaced by 200 meters from sea level to 600 meters, are labeled in meters. These surficial geology and digital elevation models are available from the USGS (San Diego Hydrogeology project; https://ca.water.usgs.gov/projects/sandiego/).

Recharge to the confined regional aquifer occurs to the east of the well sites, presumably in stream channels beginning at the foothills of the Laguna and Cuyamaca mountains, where the dominant surficial geology transitions from lower-permeability metavolcanic and igneous rocks to higher-permeability sediment. These concomitant changes in topography and geology are shown in Supplementary Figure 6 along with a box designating a probable regional recharge area, bounded by well site locations to the north, south, and west and the geologic and

topographic transitions to the east. The transitions to both low-lying topography and sediment occur ~10-15 km east of the well sites. Anders et al. (2014) hypothesized that regional recharge may originate further east at higher elevations, based on observations of lower $\delta^{18}O$ of deep, low-^{14}C samples. However, the mean $\delta^{18}O$ of the deep samples is ~1‰ lower than shallow, local Holocene recharge, which is consistent in magnitude with a glacial-interglacial climate signal, rather than an elevational signal. Indeed, a groundwater study in the western Mojave Desert also found glacial $\delta^{18}O$ values ~1‰ below Holocene $\delta^{18}O$ [20], consistent with other reconstructions of glacial-interglacial changes in precipitation $\delta^{18}O$ over southwestern United States [21]. We, therefore, suggest that recharge to the San Diego regional aquifer occurs primarily through the high permeability sediment at low elevation to the west of the onset of steep topography.

Because fine-grained sediment, which separates the shallow unconfined aquifer from the regional confined aquifer, does not occur uniformly throughout the study area, we assume that groundwater from non-artesian wells that is both younger than 5 ka (based on ^{14}C) and shallower than 150 m is part of the unconfined aquifer. Four sampled wells fit these local-recharge criteria. As evidence supporting this designation, the noble-gas reconstructed WTDs across the four samples identified as local recharge range widely (from 6 to 49 m) in a manner correlated with present-day local WTDs. We estimate representative modern local WTDs at a given well location by subtracting the groundwater elevation measured at the time of well installation from the mean surface elevation within a 1-km radius of the well. In Figure S7, replicate-mean noble gas-derived WTDs are compared to estimated mean present-day local WTD within 1-km of the well location. The large variability of these reconstructed WTDs from contemporaneous mid-Holocene groundwater contrasts with the close agreement of WTDs from contemporaneous regional groundwater.

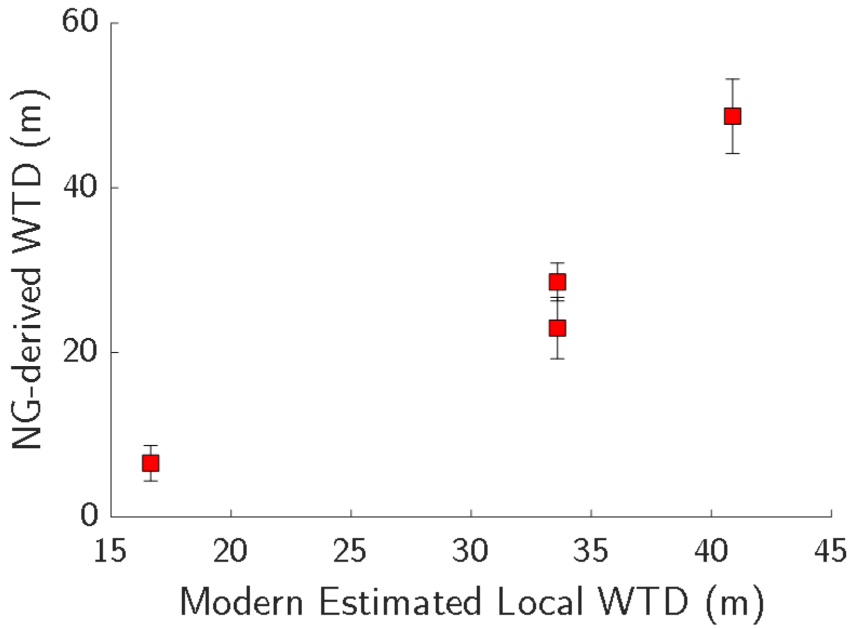

Supplementary Figure 7: Comparison of noble gas-derived WTDs to present-day estimated WTDs at the locally recharged groundwater sites. WTDs are estimated for a 1-km radius around each well by subtracting the measured water-table elevation at the time of recharge from the average surface elevation within a 1-km radius of the well. The large range of noble gas-derived WTDs among the contemporaneous mid-Holocene samples and the correlation with present-day local WTD is consistent with the assumption that this groundwater reflects a locally heterogeneous signal, not a regionally homogenous one. Error bars indicate ±2σ range of depths estimated by noble gas isotope-constrained inverse model.

For a representative comparison of regional mean WTD in the present day to reconstructed past WTDs from noble gas measurements, we analyzed output from a measurement-constrained groundwater model [22] at high resolution (~1 km x 1 km grid cells). We included all grid cells within the assumed recharge area (box in Figs. 4 and S6) except for those cells containing surface water. Unsurprisingly, single grid cells are highly dependent on local topography, such that WTD is deeper in higher elevation grid cells. Thus, there is great variability in modeled WTD over the box due to hilly topography. The mean WTD across surface-water-free grid cells (N=242) is 46.8 ± 24.7 m (1σ). Although this mean depth is distinctly lower than the ~20-m mean reconstructed WTD over the last glacial period, consistent with wetter glacial-period conditions, we caution against interpretation of the ~8 m apparent difference between the model-mean value and reconstructed Holocene-mean WTD as a meaningful result of climate change or human activities.

All San Diego groundwater samples were dated with ^{14}C of dissolved inorganic carbon (DIC), correcting for the contribution of dissolved ^{14}C-free carbonate to total DIC using a two end-member mixing model [23]. In this model, a dilution factor (q) is calculated from measured $\delta^{13}C$ ($\delta^{13}C_{meas}$), initial $\delta^{13}C$ ($\delta^{13}C_0$) and carbonate rock $\delta^{13}C$ ($\delta^{13}C_{rock}$), the latter two of which are assumed to be -25‰ and 0‰ (vs PDB).

$$q = \frac{\delta^{13}C_{meas} - \delta^{13}C_{rock}}{\delta^{13}C_0 - \delta^{13}C_{rock}} \qquad (14)$$

Radiocarbon ages (t_{14C}, in years) can then be calculated from measured modern ^{14}C fractions (a_{14C}) by:

$$t_{14C} = -8267 \ln\left(\frac{a_{14C}}{q}\right) \qquad (15)$$

and converted to calibrated age using the IntCal13 calibration curve [24]. To account for uncertainty in the mixing model assumption, we carry out 100 Monte Carlo simulations for each sample, adding normally distributed random perturbations to measured a_{14C} and $\delta^{13}C_{meas}$ of 0.1% modern carbon and 2‰, respectively. The median calibrated age from these simulations is taken as the most probable estimate of mean recharge age. However, we caution against strict interpretation of groundwater recharge age, as groundwater is best understood conceptually as a mixture of individual parcels each with different transit times since entering the saturated zone [25]. We therefore adopt ±2 ka minimum error bars on each ^{14}C age, even though the Monte Carlo age distribution is in some cases narrower. Several samples in this study also were dated with ^{81}Kr, which in all cases agreed with ^{14}C-derived ages but was of limited use given the ±~15 ka uncertainties associated with ±2-4% ^{81}Kr analytical uncertainty. One approximately ^{14}C-dead sample (SDSW1) from a 607-m deep well was found to have 41 ± 2% modern ^{81}Kr activity, consistent with an effective recharge age between ~280 and 310 ka. Interestingly, the Kr and Xe isotopes in this sample were in solubility equilibrium with atmospheric air (i.e. overlapping the square in Fig. 2), implying a WTD of zero. We hypothesize that this deep, ancient groundwater may have been directly recharged from a lake or river, without passing through an unsaturated zone, perhaps during the marine isotope stage (MIS) 8, a glacial period. Many relatively flat, inter-montane valleys are present throughout the eastern part of the study area, although they are currently dry.

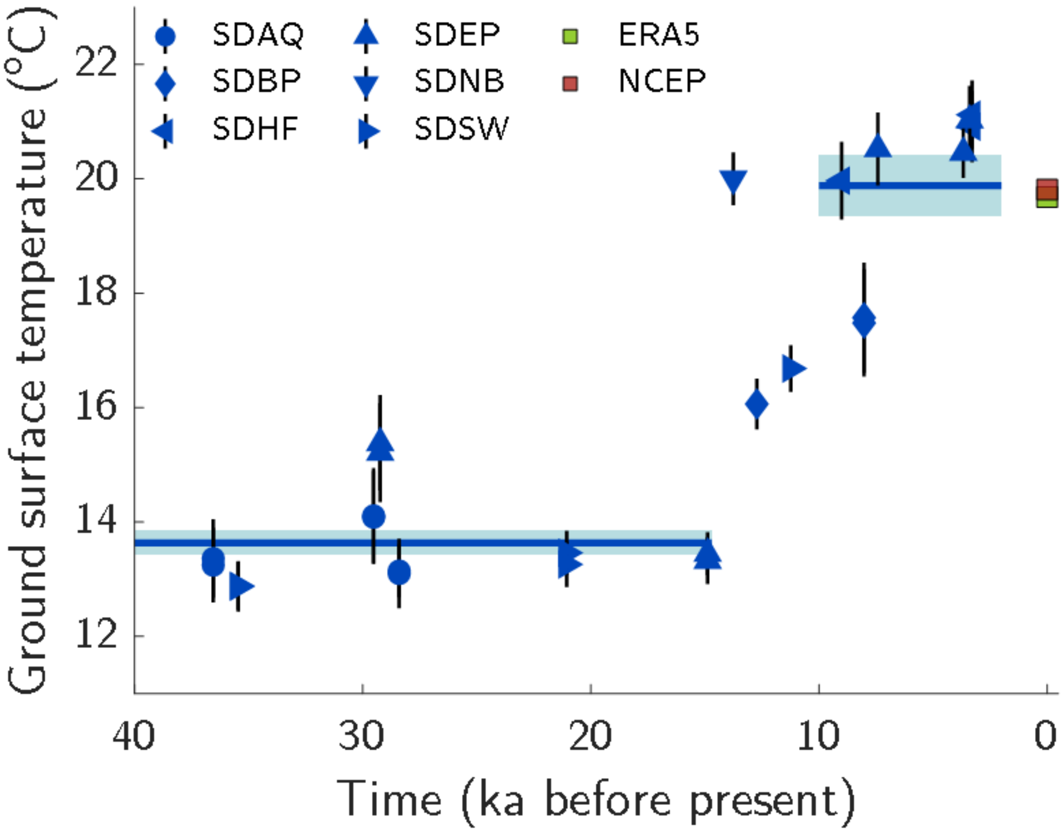

Supplementary Figure 8. Reconstructed San Diego surface temperatures since the last glacial period constrained by noble gas measurements in individual samples. Samples from well sites are identified by symbol (consistent with Figs. 4 and S6) and site name. ERA5 and NCEP reanalysis 1979-2018 mean surface soil temperatures are shown for comparison (square markers). Surface temperatures are found by subtracting the measured geothermal gradient at a well site multiplied by the reconstructed WTD. Error bars indicate ±2σ range of inversely modeled surface temperatures constrained by noble gas measurements.

As described in Supplementary Note 2, we also reconstructed mean surface temperatures from bulk noble gas concentrations, in addition to WTDs. In Supplementary Figure 8, reconstructed mean annual surface temperature is shown for all samples from the shallow and regional aquifers, except for (a) the ~300-ka old, zero-WTD sample and (b) a shallow sample with a reconstructed WTD of ~7-m, both of which are too shallow for the noble gas paleothermometry requirement of no seasonal temperature cycle at the WTD [26], and (c) glacial-period samples from the SDHF and SDBP well sites. The mean Holocene reconstructed surface temperature, 19.9 ± 0.5 °C (± 1 SE), is in close agreement with mean 1979–2018 ERA5 and NCEP reanalysis surface (0-7 cm) soil temperatures in the assumed recharge region, which are 19.7 °C and 19.8 °C, respectively. The average surface temperature during the late last glacial period (LLGP) is 13.6 ± 0.2 °C (± 1 SE), excluding the LLGP-aged samples from SDBP and SDHF sites described below. This difference in LLGP and Holocene mean surface temperatures indicates 6.2 ± 0.6 °C of deglacial warming. A warm (~20 °C) reconstructed temperature at 13.6 ka and two cooler temperatures (16-17 °C) found at 12.6 and 11.9 ka may represent recharge from the Bolling-Allerod interstadial and Younger Dryas stadial, respectively, but dating uncertainties make this speculative.

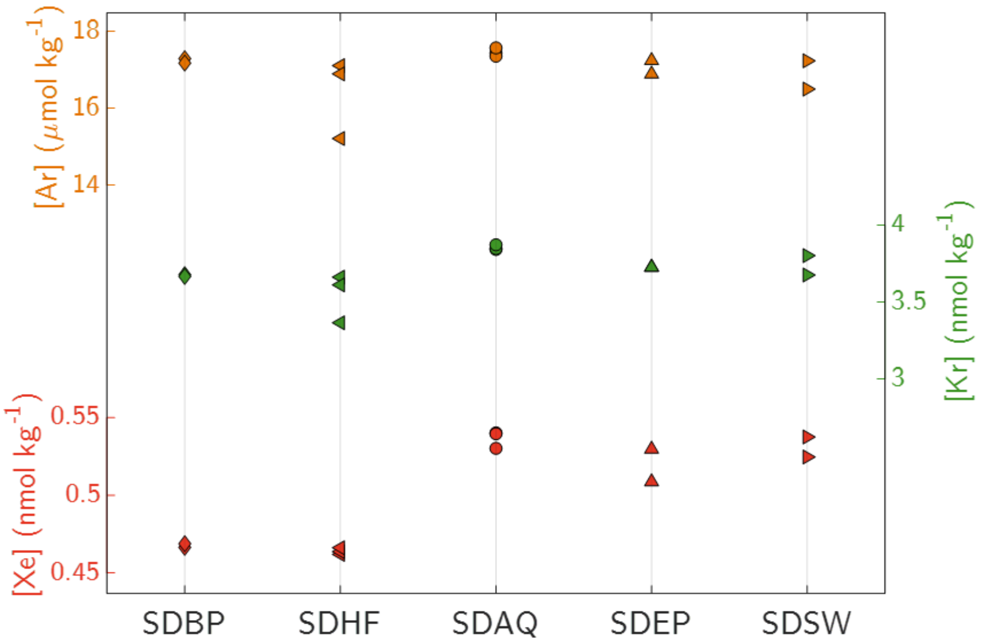

Supplementary Figure 9. Comparison of LLGP (15-40 ka recharge age) sample-mean Ar, Kr, and Xe concentrations across well sites. SDBP and SDHF samples have distinctly lower Xe concentrations and slightly lower Kr concentrations than other LLGP samples. We hypothesize that either a geothermal heat source or highly adsorbing mineral along the shared flow path may explain these low concentrations, but both theoretical considerations and isotopic observations suggest that there is no associated isotopic fractionation of these samples.

The LLGP-aged samples from well sites SDBP and SDHF (diamond and left-facing triangle markers in Figs. 4, S6, S8-9), which are located close to one another, are anomalous in their Kr and Xe concentrations. As shown in Supplementary Figure 9, the mean Ar concentrations in SDBP and SDHF LLGP samples are similar to LLGP samples from other sites (with one exception), while Kr concentrations are generally lower, and Xe concentrations are substantially lower (~12% on average). Apparent surface temperature reconstructions based on measurements in these samples cluster ~20 ºC and are thus incompatible with the 13-14 ºC LLGP temperatures consistently observed at other sites as well as with our climatological understanding of the LLGP. We note that the single SDHF sample (SDHF3, replicated twice) with low [Ar] is the same ~24 ka sample with a deep (~45 m) WTD, which may be linked to the reported glacial "megadrought" in Southern California around this time [27–29]. Thus, the low [Ar] may indicate low excess air in this sample, since excess air has been suggested to correlate inversely with WTD [30,31]. However, the preservation of such a megadrought signal despite dispersive mixing would require that the climatic period was long-lived (several thousand years) or that dispersivity was low. We cannot therefore conclude that this sample reflects a real climate signal rather than a hydrogeological one. This sample also appears lower than other LLGP SDHF and SDBP samples in [Kr], although by a lesser relative amount than for [Ar], but is virtually indistinguishable from other SDBP and SDHF samples in terms of [Xe]. Because the impact of excess air is largest for less soluble gases, this pattern (largest differences for [Ar], smallest for [Xe]) supports the notion that this sample has relatively low excess air compared to other LLGP samples and is consistent with its deep reconstructed WTD.

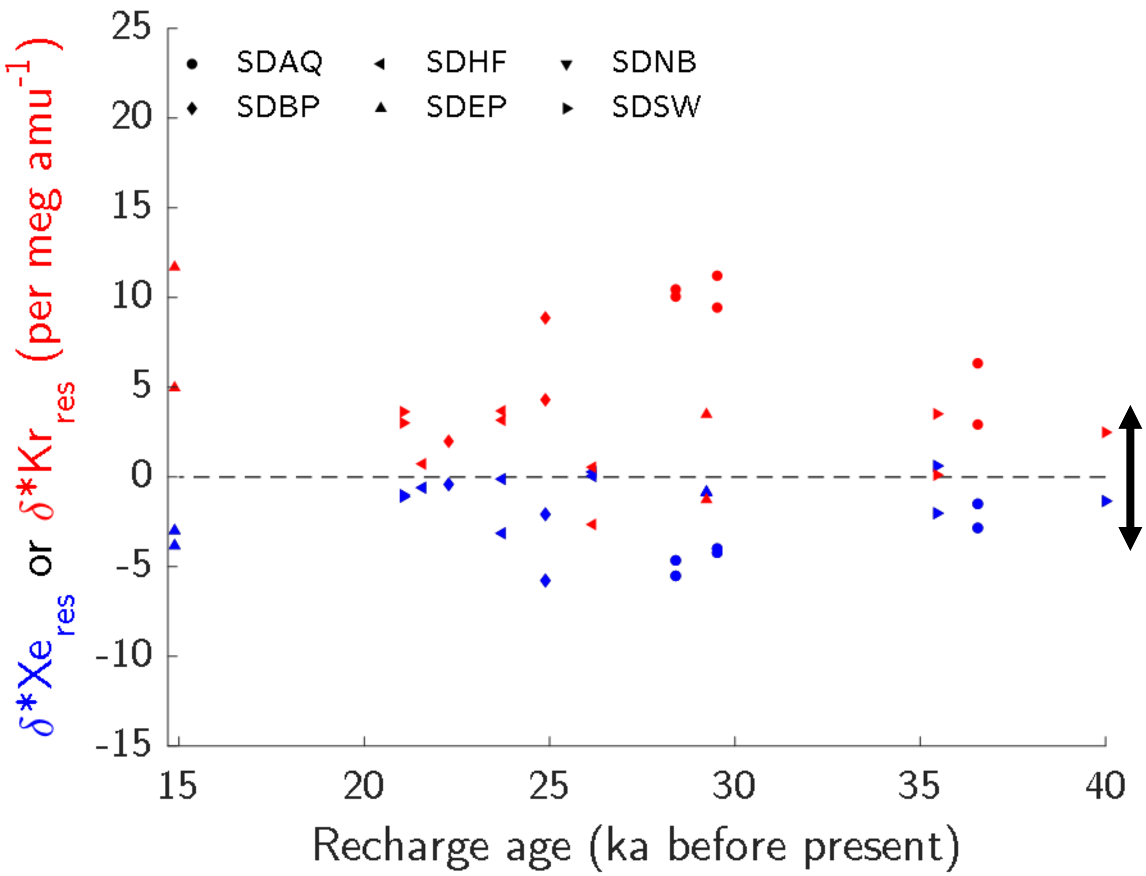

Supplementary Figure 10. Isotopic measurement-model residuals from inverse model fitting of LLGP groundwater samples. The Xe and Kr isotope residuals from SDBP and SDHF samples that display low Kr and Xe concentrations are not distinct from other LLGP samples, implying that the process responsible for the low Kr and Xe did not cause isotopic fractionation. Arrows indicate ±1-m of gravitational settling fractionation for context.

We offer two hypotheses for the low Kr and Xe concentrations in these samples. First, because these sites are located close together and along an east-west line parallel to groundwater flow, it is possible that a source of geothermal heat along their shared flow path was present during the LLGP. In this case, these noble gas concentrations would reflect the true recharge temperatures, affected by an anomalous local geothermal feature. A second hypothesis is that a strongly adsorbing mineral along their shared flow path removed Kr and Xe. Xe and, to a lesser extent, Kr, are known to be susceptible to adsorption [32,33], which has been demonstrated to be independent of any isotopic fractionation [34,35]. This adsorption would cause a warm bias in reconstructed temperature. Although we must reject the noble gas temperatures derived from the LLGP SDBP and SDHF samples as being representative of regional conditions, we note that any influence of either a geothermal heat source or adsorption on isotopic composition is small relative to the contribution of gravitational settling. For example, the sensitivity of Kr and Xe isotopic solubility fractionation to temperature is extremely small (<0.25 per meg amu^{-1} $^{o}C^{-1}$), such that even a 10 ^{o}C change in recharge temperature is equivalent to less than one meter of gravitational settling fractionation (4.0 per meg amu^{-1}). Similarly, excess air fractionation and thermal diffusion fractionation are equivalent in magnitude to only single meters of gravitational settling fractionation (Fig 2). As further evidence that isotopic ratios are not affected by the

process responsible for the low Kr and Xe in LLGP SDBP and SDHF samples, δ*Kr and δ*Xe residuals from these samples are indistinguishable from other LLGP samples (Supplementary Figure 10). Similarly, WTDs reconstructed from these samples closely match those from other LLGP sites. Thus, while we have insufficient evidence to determine which, if either, of these two proposed hypotheses is correct, it is highly probable that the Kr and Xe isotopic composition of these samples, relative to gravitational settling fractionation at the meter scale, is insensitive to whichever process is responsible for the low Kr and Xe concentrations.

Supplementary Note 5. Mojave Desert Groundwater Study: Detailed Description

A total of 18 groundwater samples were collected over a large spatial extent (from 34.4 to 35.0 ^{o}N and from 117.4 to 118.1 ^{o}W) of the western Mojave Desert. These samples were collected from 11 total supply wells, unlike the narrow-screened monitoring wells from which the San Diego samples were collected. These supply wells were each screened over large depth intervals, potentially allowing for mixing of waters of different recharge age. We do not attempt to interpret these samples in a paleoclimatic context.

As described in the main text and shown in Figs. 2 and S3, the Mojave groundwater samples exhibit a discordant δ*Kr vs δ*Xe relationship from the San Diego and Fresno samples, which fall close to the predicted line for isotopic fractionation due only to gravitational settling and solubility fractionation. This Mojave-specific feature manifests itself as positive δ^*Kr_{res} and negative δ^*Xe_{res} when fitting measurements to the WTD/temperature model. In other words, individual samples plot above the expected fractionation line for gravitational settling and solubility fractionation on a plot of δ*Kr vs δ*Xe (Supplementary Figure 11). This isotopic feature is most pronounced for samples with higher δ*Kr and δ*Xe, which yield deeper apparent WTDs. We can think of two candidate mechanisms that are not accounted for in our UZ air fractionation model but which have been observed before in porous media and found to fractionate noble gases in manner consistent with our Kr and Xe isotopic observations.

The first candidate process is steady-state kinetic fractionation of noble gases driven by a diffusive atmosphere-to-UZ flux of O_2 in response to O_2 consumption in the deep UZ. The so-called "O_2 depletion" (OD) model was previously introduced to account for anomalously low reconstructed noble gas temperatures caused by elevated noble gas partial pressures above the water table [36]. In this model, steady-state consumption of O_2 in the deep UZ, leads to non-equimolar replacement in UZ air by CO_2 (because CO_2 is highly soluble), such that the sum of partial pressures of O_2 and CO_2 decreases. Because total UZ air pressure remains constant, the deficit of (O_2 + CO_2) from deep UZ air leads to advective replacement by other dry air constituents, such that the partial pressure of noble gases in UZ air increases directly with (O_2 + CO_2) deficit. Decreases in the (O_2 + CO_2) mole fraction in UZ air of several percent (note: dry air has an O_2 + CO_2 mole fraction of ~21%) and associated increases in noble gas partial pressures have been observed in several studies [37,38]. If a decrease in (O_2 + CO_2) partial pressure and corresponding increase in noble gas partial pressures in deep UZ air is maintained at steady state, the partial pressure gradient between noble gases in deep UZ air and surface air will drive an upwards diffusive flux against downward diffusion of O_2, causing kinetic isotopic fractionation of noble gases. This kinetic fractionation can be thought of as an inverse analog to water-vapor flux fractionation [9], such that steady-state diffusion of noble gases out of the UZ leads to an increase in heavy-to-light isotope ratios in UZ air relative to the atmosphere due to the higher diffusivity of the lighter isotope.

We can model this isotopic fractionation (ε_{O2}) using an equation analogous to Supplementary Equation 12, making use of the approximation given in Severinghaus et al. (1996):

$$\varepsilon_{O2} \approx \left(\frac{D_{h\text{-}O2}}{D_{l\text{-}O2}} - 1\right)\Delta X_{O2} \qquad (16)$$

where ΔX_{O2} is the mole fraction difference in (O_2 + CO_2) between UZ air and atmospheric air (negative if the UZ air mole fraction is lower), and $\frac{D_{h\text{-}O2}}{D_{l\text{-}O2}}$ is the ratio of binary diffusivities against O_2 for a heavy and light isotope of a noble gas [10]. Normalized by isotopic mass difference $\frac{D_{h\text{-}O2}}{D_{l\text{-}O2}} - 1$ is greater for Kr isotope ratios (-1.6‰ amu^{-1}) than for Xe isotope ratios (-0.7‰ amu^{-1}). Thus, for negative ΔX_{O2}, isotopic fractionation is positive (increase of heavy isotopes vs light isotopes) and affects Kr isotopes more strongly than Xe isotopes. In Supplementary Figure 11, expected δ*Kr and δ*Xe fractionation is shown for ΔX_{O2} = -0.5%. One can see that O_2 depletion fractionation acting in addition to gravitational settling and solubility fractionation would cause dissolved Kr and Xe isotope to plot above the gravity/solubility line in Supplementary Figure 11, consistent with the Mojave observations. In this case, WTDs would be overestimated by our model because oxygen depletion, like gravitational settling, leads to elevated δ*Kr and δ*Xe. The fact that the predicted magnitude of this increase is not 1:1 for δ*Kr and δ*Xe, unlike gravitational settling, could explain the positive δ^*Kr_{res} and negative δ^*Xe_{res} we observe in the Mojave Desert samples (Supplementary Figure 3).

The second candidate process, which we call "kinetic disequilibrium" fractionation, is caused by storm-driven barometric pumping of the UZ. Kinetic disequilibrium acts against gravitational settling by lowering both δ*Kr and δ*Xe in different proportions, such that δ*Xe is decreased more than δ*Kr. In the envisioned scenario, barometric pumping of the UZ initially disrupts gravitational settling by effectively mixing gravitationally enriched air with unfractionated atmospheric air (δ=0). Then, once pumping has ceased, UZ air composition is driven back towards gravitational equilibrium by molecular diffusion. Because Xe is slower diffusing than Kr, and because light isotopes are faster diffusing than heavy isotopes, there is kinetic fractionation that affects the isotope ratios of Kr and Xe differently. If the characteristic timescale between barometric pumping events is shorter than the timescale for molecular diffusion to return the Kr and Xe isotopic composition of deep UZ air back to the expected steady-state composition (driven only by gravitational settling, thermal diffusion, and water-vapor flux fractionation) then the isotopic signal of kinetic disequilibrium will persist and be transmitted to groundwater via dissolution at the water table.

Barometric pumping fractionation has been observed in polar firn air [12], and a 2-D firn air model (Birner et al., 2018) has simulated the combined effects of layering and barometric pumping to determine the isotopic mass-difference normalized sensitivities of Kr and Xe to kinetic disequilibrium. This simulation would suggest that δ*Kr and δ*Xe would both decrease due to barometric pumping, with δ*Xe affected 39% more than δ*Kr, as shown in Supplementary Figure 11. If this process is responsible for the deviation of Mojave Desert samples from the gravity/solubility expectation line, then our inverse model would underestimate true WTD and yield negative δ^*Xe_{res} and positive δ^*Kr_{res}, again consistent with our observations (Supplementary Figure 3).

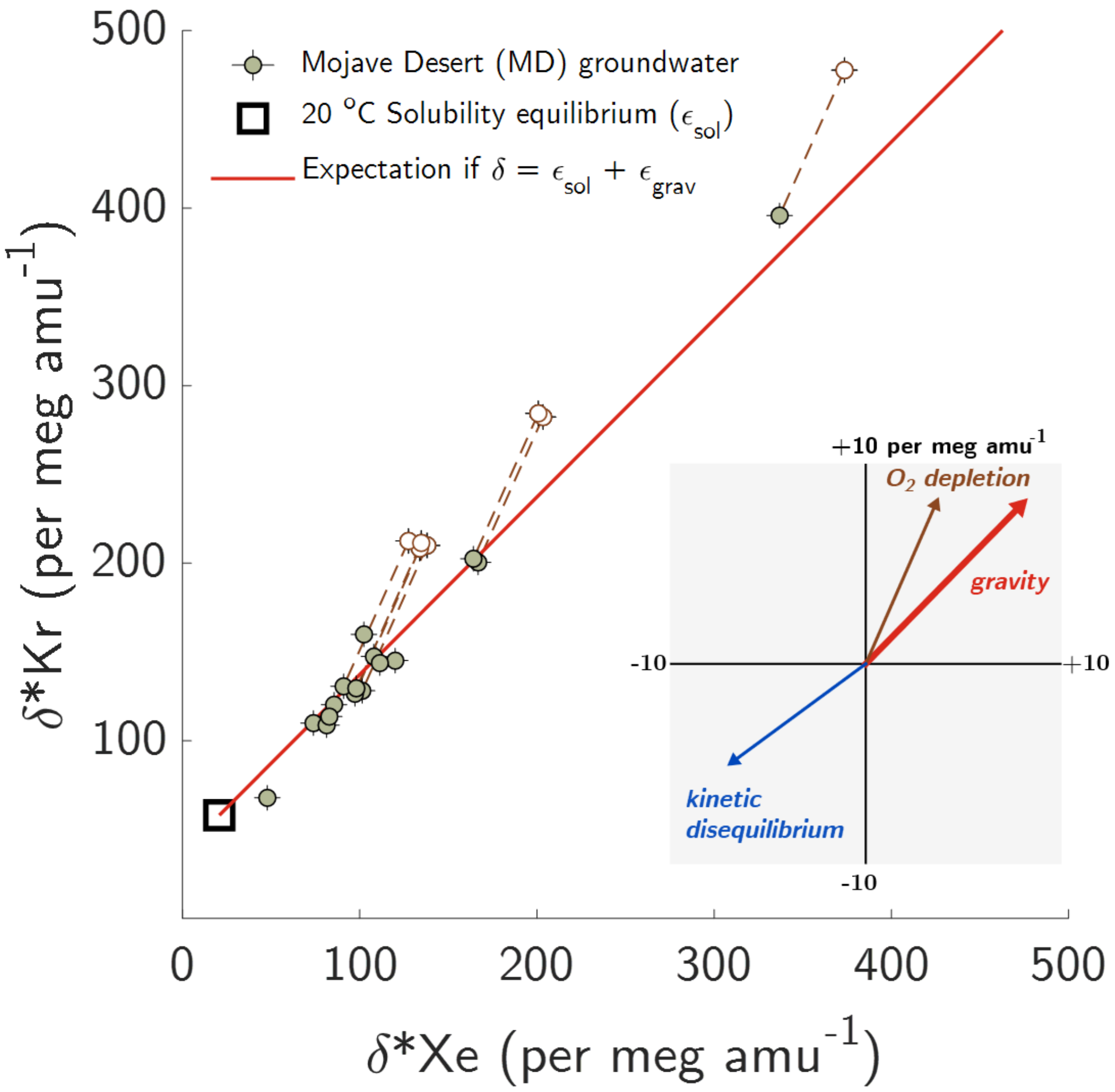

Supplementary Figure 11. Mojave Desert δ*Kr and δ*Xe (reproduced from Fig. 2) compared to the expectation that isotopic fractionation is governed solely by solubility fractionation and gravitational settling in UZ air prior to dissolution. A possible correction is shown for 5% oxygen depletion ($\Delta X_{O2} = -0.05$), bringing several uncorrected values (open circles) in closer agreement with the expected trendline. Shown in the inset are Kr and Xe isotopic fractionation associated with (a) 2 m of gravitational settling, (b) 0.5% O_2 depletion ($\Delta X_{O2} = -0.005$), and (c) kinetic disequilibrium due to barometric pumping (-5per meg amu^{-1} for δ*Kr).

Although we cannot conclusively differentiate between these two possible explanations for the Mojave Desert Kr and Xe isotope observations, the apparent sensitivity of Mojave δ^*Xe_{res} and δ^*Kr_{res} to WTD is consistent with fractionation due to molecular diffusion in UZ air. The timescale for diffusive re-equilibration increases quadratically with depth (i.e. the e-folding timescale for 1-D diffusive re-equilibration, τ, is equal to z^2/D, where z is UZ depth and D is molecular diffusivity). In shallow UZ air, repeated barometric pumping or O_2 consumption may cause short-lived fractionation of UZ air that quickly returns to the expected ε_{UZ} predicted by our (mostly gravitational) steady-state UZ air model. In other words, the timescale of diffusive re-equilibration may be shorter than the timescales of O_2 consumption and barometric pumping for shallower UZ air. However, in deep UZ air, the timescale of O_2 consumption or barometric

pumping events maybe shorter than the diffusive-equilibration timescale, leaving behind a signal of kinetic fractionation in the steady-state UZ air composition above the WTD, which ultimately dissolves into groundwater. In extreme cases, such as these Mojave Desert samples, this can lead to over or under estimation of ~100-m deep WTDs by 10-20 m based on Kr and Xe isotopic composition. In principle, Ar isotopes and noble gas temperatures could help identify which mechanism is at play and, ideally, provide a correction for more robust WTD determination. However, $^{40}/_{36}$Ar is affected by β decay of ^{40}K and $^{38}/_{36}$Ar is measured at ~10 per meg amu^{-1} precision (±1σ) and is significantly more sensitive to solubility fractionation, excess air, thermal diffusion, and water-vapor flux fractionation, rendering it difficult to conclusively identify an anomalous fractionation signal associated with O_2 depletion or kinetic disequilibrium. We suggest that Ne isotope measurements at high precision in future studies may provide a useful clue, as $\delta^{22}/_{20}$Ne is negligibly affected by kinetic disequilibrium (because Ne is fast diffusing relative to Kr and Xe) but strongly affected by O_2 depletion ($\frac{D_{h\text{-}O2}}{D_{l\text{-}O2}} - 1$ is -14‰ amu^{-1} for $\delta^{22}/_{20}$Ne, 20 times larger than for δ*Xe). The noble gas-derived mean annual surface temperature in one deep-WTD Mojave sample of LLGP age did appear extremely low (~10 ºC), lower than the expected LLGP mean surface temperature (~4-5 ºC below present day ~17 ºC MAST) and is therefore in principle consistent with the O_2 depletion hypothesis. However, this temperature estimate is too underconstrained to meaningfully draw any conclusions about which isotopic fractionation mechanism may be at play. We suggest that future dissolved isotopic analyses, including Ne isotopes, may provide evidence in support of either of these candidate mechanisms.

Supplementary References

1. Petrenko, V. V., Severinghaus, J. P., Brook, E. J., Reeh, N. & Schaefer, H. Gas records from the West Greenland ice margin covering the Last Glacial Termination: a horizontal ice core. *Quat. Sci. Rev.* **25**, 865–875 (2006).
2. COESA. *U.S. Standard Atmosphere*. (1976).
3. Seltzer, A. M., Severinghaus, J. P., Andraski, B. J. & Stonestrom, D. A. Steady state fractionation of heavy noble gas isotopes in a deep unsaturated zone. *Water Resour. Res.* **53**, 2716–2732 (2017).
4. Seltzer, A. M., Ng, J. & Severinghaus, J. P. Precise determination of Ar, Kr and Xe isotopic fractionation due to diffusion and dissolution in fresh water. *Earth Planet. Sci. Lett.* **514**, 156–165 (2019).
5. Aeschbach-Hertig, W., Peeters, F., Beyerle, U. & Kipfer, R. Palaeotemperature reconstruction from noble gases in ground water taking into account equilibration with entrapped air. *Nature* **405**, 1040–4 (2000).
6. Aeschbach-Hertig, W., El-Gamal, H., Wieser, M. & Palcsu, L. Modeling excess air and degassing in groundwater by equilibrium partitioning with a gas phase. *Water Resour. Res.* **44**, (2008).
7. Jenkins, W. J., Lott, D. E. & Cahill, K. L. A determination of atmospheric helium, neon, argon, krypton, and xenon solubility concentrations in water and seawater. *Mar. Chem.* **211**, 94–107 (2019).
8. Schwander, J. The transformation of snow to ice and the occlusion of gases. in *The Environmental Record in Glaciers and Ice Sheets* (eds. Oeschger, H. & Langway, C. C.) 53–67 (Wiley, 1989).
9. Severinghaus, J. P., Bender, M. L., Keeling, R. F. & Broecker, W. S. Fractionation of soil gases by diffusion of water vapor, gravitational settling, and thermal diffusion. *Geochim.*

Cosmochim. Acta **60**, 1005–1018 (1996).
10. Fuller, E. N., Schettler, P. D. & Giddings, J. C. A new method for prediction of binary gas-phase diffusion coefficients. *Ind. Eng. Chem.* **16**, 551 (1966).
11. Grachev, A. M. & Severinghaus, J. P. Determining the thermal diffusion factor for 40Ar/36Ar in air to aid paleoreconstruction of abrupt climate change. *J. Phys. Chem. A* **107**, 4636–4642 (2003).
12. Kawamura, K. *et al.* Kinetic fractionation of gases by deep air convection in polar firn. *Atmos. Chem. Phys.* **13**, 11141–11155 (2013).
13. Saraceno, J., Kulongoski, J. T. & Mathany, T. M. A novel high-frequency groundwater quality monitoring system. (2018). doi:10.1007/s10661-018-6853-6
14. University, C. for I. E. S. I. N.-C.-C. Gridded Population of the World, Version 4 (GPWv4): Population Density, Revision 11. (2018).
15. Bollhöfer, A. *et al.* Half a century of Krypton-85 activity concentration measured in air over Central Europe: Trends and relevance for dating young groundwater. *J. Environ. Radioact.* **205**–**206**, 7–16 (2019).
16. Bullister, J. L., Wisegarver, D. P. & Menzia, F. A. The solubility of sulfur hexafluoride in water and seawater. *Deep Sea Res. Part I Oceanogr. Res. Pap.* **49**, 175–187 (2002).
17. Kip Solomon, D., Cook, P. G. & Sanford, W. E. Dissolved Gases in Subsurface Hydrology. *Isot. Tracers Catchment Hydrol.* 291–318 (1998). doi:10.1016/B978-0-444-81546-0.50016-1
18. Schlosser, P., Stute, M., Sonntag, C. & Otto Münnich, K. Tritiogenic 3He in shallow groundwater. *Earth Planet. Sci. Lett.* **94**, 245–256 (1989).
19. Anders, R., Mendez, G. O., Futa, K. & Danskin, W. R. A Geochemical Approach to Determine Sources and Movement of Saline Groundwater in a Coastal Aquifer. *Groundwater* **52**, 756–768 (2014).
20. Kulongoski, J. T., Hilton, D. R., Izbicki, J. A. & Belitz, K. Evidence for prolonged El Nino-like conditions in the Pacific during the Late Pleistocene: a 43ka noble gas record from California groundwaters. *Quat. Sci. Rev.* **28**, 2465–2473 (2009).
21. Jasechko, S. *et al.* Late-glacial to late-Holocene shifts in global precipitation δ^{18}O. *Clim. Past* **11**, 1375–1393 (2015).
22. Fan, Y., Li, H. & Miguez-Macho, G. Global Patterns of Groundwater Table Depth. *Science.* **339**, 940–943 (2013).
23. Pearson, F. J. J. & Hanshaw, B. B. Sources of Dissolved Carbonate Species in Groundwater and their Effects on Carbon-14 Dating. in *Isotopes in Hydrology 1970* 271–285 (International Atomic Energy Agency, 1970).
24. Reimer, P. J. *et al.* IntCal13 and Marine13 Radiocarbon Age Calibration Curves 0–50,000 Years cal BP. *Radiocarbon* **55**, 1869–1887 (2013).
25. Bethke, C. M. & Johnson, T. M. Groundwater Age and Groundwater Age Dating. *Annu. Rev. Earth Planet. Sci.* **36**, 121–152 (2008).
26. Stute, M. & Schlosser, P. Principles and Applications of the Noble Gas Paleothermometer. in *Climate Change in Continental Isotopic Records, Volume 78* (eds. Savin, P. K., C., S. K., J., L. & S., M.) 89–100 (American Geophysical Union (AGU), 1993). doi:10.1029/gm078p0089
27. Kirby, M. E. *et al.* A late Wisconsin (32–10k cal a BP) history of pluvials, droughts and vegetation in the Pacific south-west United States (Lake Elsinore, CA). *J. Quat. Sci.* **33**, 238–254 (2018).

28. Heusser, L. E., Kirby, M. E. & Nichols, J. E. Pollen-based evidence of extreme drought during the last Glacial (32.6-9.0 ka) in coastal southern California. *Quat. Sci. Rev.* **126**, 242–253 (2015).
29. Feakins, S. J., Wu, M. S., Ponton, C. & Tierney, J. E. Biomarkers reveal abrupt switches in hydroclimate during the last glacial in southern California. *Earth Planet. Sci. Lett.* **515**, 164–172 (2019).
30. Heaton, T. H. E. & Vogel, J. C. "Excess air" in groundwater. *Journal of Hydrology* **50**, 201–216 (1981).
31. Ingram, R. G. S., Hiscock, K. M. & Dennis, P. F. Noble gas excess air applied to distinguish groundwater recharge conditions. *Environ. Sci. Technol.* **41**, 1949–55 (2007).
32. Podosek, F. A., Bernatowicz, T. J. & Kramer, F. E. Adsorption of xenon and krypton on shales. *Geochim. Cosmochim. Acta* **45**, 2401–2415 (1981).
33. Yang, J., Lewis, R. S. & Anders, E. Sorption of noble gases by solids, with reference to meteorites. I. Magnetite and carbon. *Geochim. Cosmochim. Acta* **46**, 841–860 (2003).
34. Marrocchi, Y. & Marty, B. Experimental determination of the xenon isotopic fractionation during adsorption. *Geophys. Res. Lett.* **40**, 4165–4170 (2013).
35. Bernatowicz, T. J. & Podosek, F. A. Adsorption and isotopic fractionation of Xe. *Geochim. Cosmochim. Acta* **50**, 1503–1507 (1986).
36. Hall, C. M., Castro, M. C., Lohmann, K. C. & Ma, L. Noble gases and stable isotopes in a shallow aquifer in southern Michigan: Implications for noble gas paleotemperature reconstructions for cool climates. *Geophys. Res. Lett.* **32**, n/a-n/a (2005).
37. Hall, C. M., Castro, M. C., Lohmann, K. C. & Sun, T. Testing the noble gas paleothermometer with a yearlong study of groundwater noble gases in an instrumented monitoring well. *Water Resour. Res.* **48**, (2012).
38. Freundt, F., Schneider, T. & Aeschbach-Hertig, W. Response of noble gas partial pressures in soil air to oxygen depletion. *Chem. Geol.* **339**, 283–290 (2013).
39. Birner, B., Buizert, C., Wagner, T. J. W. & Severinghaus, J. P. The influence of layering and barometric pumping on firn air transport in a 2-D model. *Cryosph.* **12**, (2018).
