# Supplementary material for: Deglacial water-table decline in Southern California recorded by noble gas isotopes
Source: Nat Commun. 2019 Dec 16;10:5739. doi: 10.1038/s41467-019-13693-2 (PMC6915717; doi:10.1038/s41467-019-13693-2)
Supplement: Supplementary file 3 — Description of Additional Supplementary Files [file 41467_2019_13693_MOESM3_ESM.pdf]

Description of Additional Supplementary Files

File name: Supplementary Data 1
Description: 667 Noble gas isotope ratio and bulk concentration measurements for all 58 groundwater samples 668 presented in this study; sample metadata (well information and hyperlinks to USGS well data, 669 carbon isotopes, 14C ages); inverse model results (water table depths, noble gas temperatures)
